# From biomarkers to intervention: Translational potential of endogenous retrovirus‐derived chimeric RNAs

**DOI:** 10.1002/ctm2.70834

**Published:** 2026-09-16

**Authors:** Ziyi Ouyang, Hongqing Liang, Dan Zhang

**Affiliations:** ^1^ Institute of Medical Genetics and Development Key Laboratory of Reproductive Genetics (Ministry of Education), Department of Reproductive Endocrinology Women's Hospital School of Medicine Zhejiang University Hangzhou China; ^2^ Zhejiang Provincial Birth Defect Control and Prevention Research Center, and Zhejiang International Science and Technology Cooperation Base of Reproductive Health Hangzhou China

1

Early human embryonic development depends on successful completion of several critical developmental milestones. Among these, zygotic genome activation (ZGA) is a pivotal process during which embryos initiate their own transcription program, transitioning from maternal transcript dependence to embryonic gene expression.^1^ Successful execution of ZGA is essential for subsequent developmental events. However, a notable proportion of in vitro fertilisation (IVF)‐derived embryos experience unexplained developmental delay or arrest during this critical window, which significantly complicates the selection of suitable embryos for transfer.^2^ Current embryo assessment and selection criteria primarily rely on morphological grading and cleavage morpho‐kinetics obtained from time‐lapse imaging.^3^ Although these approaches provide valuable phenotypic information, they cannot directly evaluate the functional competence of the transcription network required for successful ZGA and subsequent development.^4^ Thus, molecular markers that genuinely reflect the functional state of ZGA represent a promising and biologically grounded target for developing clinically relevant assays.

Our recent study identified endogenous retrovirus (ERV), MLT2A1, derived chimeric RNAs as critical regulators ensuring successful ZGA in human early embryos.^5^ Specifically, we found that MLT2A1 can be transcribed together with downstream sequences, engendering a diverse pool of chimeric RNAs. These chimeric RNAs share a common 5′ MLT2A1 sequence but contain variable 3′ fusion sequences, which include both protein‐coding and non‐coding regions. The diversity of these 3′ sequences expands the genomic targeting capacity of MLT2A1 RNAs, enabling them to bind different ZGA genes or regulatory elements. In parallel, the 5′ MLT2A1 region recruits heterogeneous nuclear ribonucleoprotein U (HNRNPU) and RNA polymerase II to promote transcriptional activation. Collectively, these chimeric RNAs establish a self‐reinforcing regulatory network that ensures robust activation of global ZGA genes. Notably, MLT2A1 expression is reduced in human IVF embryos arrested at the eight‐cell stage, and experimental depletion of MLT2A1 disrupts ZGA gene transcription and impairs embryo development. Together, these findings demonstrate that upregulation of MLT2A1 at ZGA is not merely correlated with this process but is functionally required for its successful completion in human embryos.

The functional importance of MLT2A1, together with its clinical association with developmentally arrested eight‐cell embryos, suggests that MLT2A1‐derived chimeric transcripts may serve as indicators of the underlying ZGA transcriptional network. Integrating such molecular information with conventional morphological assessment may improve the evaluation of an embryo's capacity to successfully navigate ZGA. Given the high copy number and sequence diversity of these chimeric RNAs, clinical implementation could feasibly focus on a selected subset of development‐associated copies or their fusion sequences, using targeted low‐input RNA assays. Nevertheless, these molecular indicators should not yet be regarded as surrogate endpoints, as the capacity to complete ZGA and subsequent pre‐implantation development does not guarantee implantation or pregnancy, which depend on additional layers of regulation beyond the ZGA program.

From a translational perspective, current assays that rely on embryo biopsy remain invasive.^6^ A clinically deployable approach for detecting MLT2A1 and other ERV RNAs would therefore ideally incorporate a non‐invasive sampling strategy. A critical first step is to determine whether MLT2A1 RNAs or other ERV‐derived RNAs could be reproducibly detected on the cell surface, blastocoelic fluid or in spent embryo culture medium, and more importantly, whether these low‐abundance signals accurately reflect the intraembryonic transcriptional activity or developmental status. Validation in independent IVF cohorts will be required to establish whether ERV‐derived RNA detection provides prognostic information beyond morphological and morpho‐kinetics parameters. Technical challenges must also be accounted for, including low‐input RNA measurements and accurate assignment of sequencing reads to repetitive genomic regions.^7^ These issues are particularly relevant to ERV‐derived RNAs, as minor variations in library preparation, amplification or mapping strategy can skew the detection of individual RNA copies. Furthermore, the biological causes of embryonic arrest are heterogeneous, and ERV signatures should not be expected to account for all cases of developmental failure. Chromosomal abnormalities, mitochondrial dysfunction and defects in maternal factors may independently converge on similar arrest phenotypes.^2^ It will therefore be important to determine whether aberrant ERV marker expression defines a distinct molecular subset of arrested embryos, rather than serving as a broad indicator of general developmental incompetence.

Beyond their utility as biomarkers, the active role of MLT2A1s towards ZGA regulation also implies that therapeutic manipulation of their expression could reshape embryo developmental potential. Indeed, our data indicate that microinjection of chimeric RNAs derived from selected MLT2A1 copies can partially restore ZGA gene expression.^5^ However, direct manipulation, whether via genome editing or exogenous RNA microinjection, could pose unpredictable developmental risks. In addition, given the widespread distribution and pleiotropic transcriptional effects of ERV‐derived RNAs, targeting the large number of ERV copies within a given subfamily presents a considerable challenge. Therefore, rather than manipulating ERV transcripts, a more feasible strategy may lie in identifying and modulating their upstream regulatory signals or pathways that govern their expression.

Because ERV activation is tightly regulated by epigenetic status, metabolic perturbations or alterations in in vitro culture conditions may influence ERV‐derived RNA expression through epigenetic remodelling.^8^, ^9^ Maternal metabolic stresses, such as those associated with obesity and diabetes, are prominent drivers of epigenetic remodelling in oocytes or early embryos, and are well‐established contributors to reproductive failure and developmental abnormalities.^10^ However, whether these upstream disturbances significantly alter ERV‐derived chimeric transcript expression to compromise early embryo developmental potential remains to be validated. Therefore, investigating whether defective ERV activation underlies pregnancy failure induced by these external stresses is of paramount importance. Rather than directly targeting ERV transcripts, designing intervention strategies to modulate maternal metabolic or environmental conditions, thereby remodelling ERV expression, represents a more practically achievable approach. Furthermore, changes in ERV‐derived chimeric RNAs could serve as an early indicator of whether these metabolic or culture‐based interventions are effectively restoring normal ZGA or developmental programs. Elucidating how ERVs respond to upstream environmental signals or maternal pre‐pregnancy status will not only deepen our understanding of ERV function during early embryogenesis, but also enhance their clinical application.

Our work reveals novel and critical roles of ERVs that underscore their translational potential (Figure 1). Further studies are needed to determine whether ERVs can indicate the embryonic transcriptional program required for sustained development, and whether ERV‑derived RNAs may eventually guide the design of developmental interventions. Overall, ERV‑derived chimeric RNAs represent a paradigm shift in the understanding and prediction of human embryo competence in future clinical practice.

**FIGURE 1:**
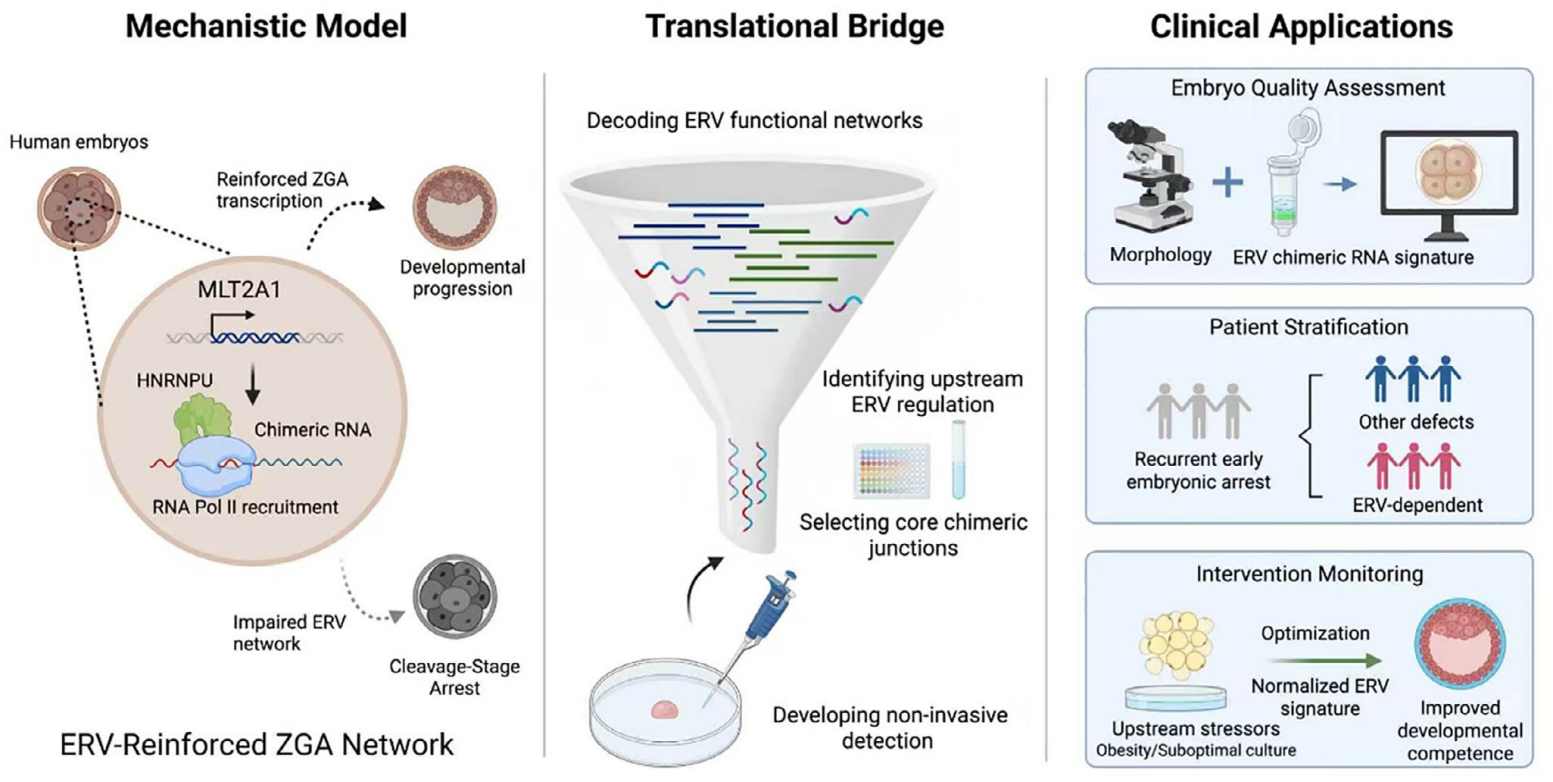
Functional roles of endogenous retrovirus (ERV), the translation bridges and the clinical strategies for utilising ERV‐derived chimeric RNAs in human reproductive practice.

## AUTHOR CONTRIBUTIONS

Z.O.Y. wrote the first draft; H.L. and D.Z. organized the review structure and supervised the writing.

## CONFLICT OF INTEREST STATEMENT

The authors declare they have no conflicts of interest.

## ETHICS STATEMENT

Not applicable.

## Data Availability

Data sharing is not applicable to this article as no new data were created or analysed in this study.
